# Supplementary figures and images for: Evaluation of near-infrared spectroscopy as a contactless method for health monitoring of resin-based coating materials applied to concrete surfaces
Source: PLoS One. 2023 Jun 28;18(6):e0287918. doi: 10.1371/journal.pone.0287918 (PMC10306184; doi:10.1371/journal.pone.0287918)

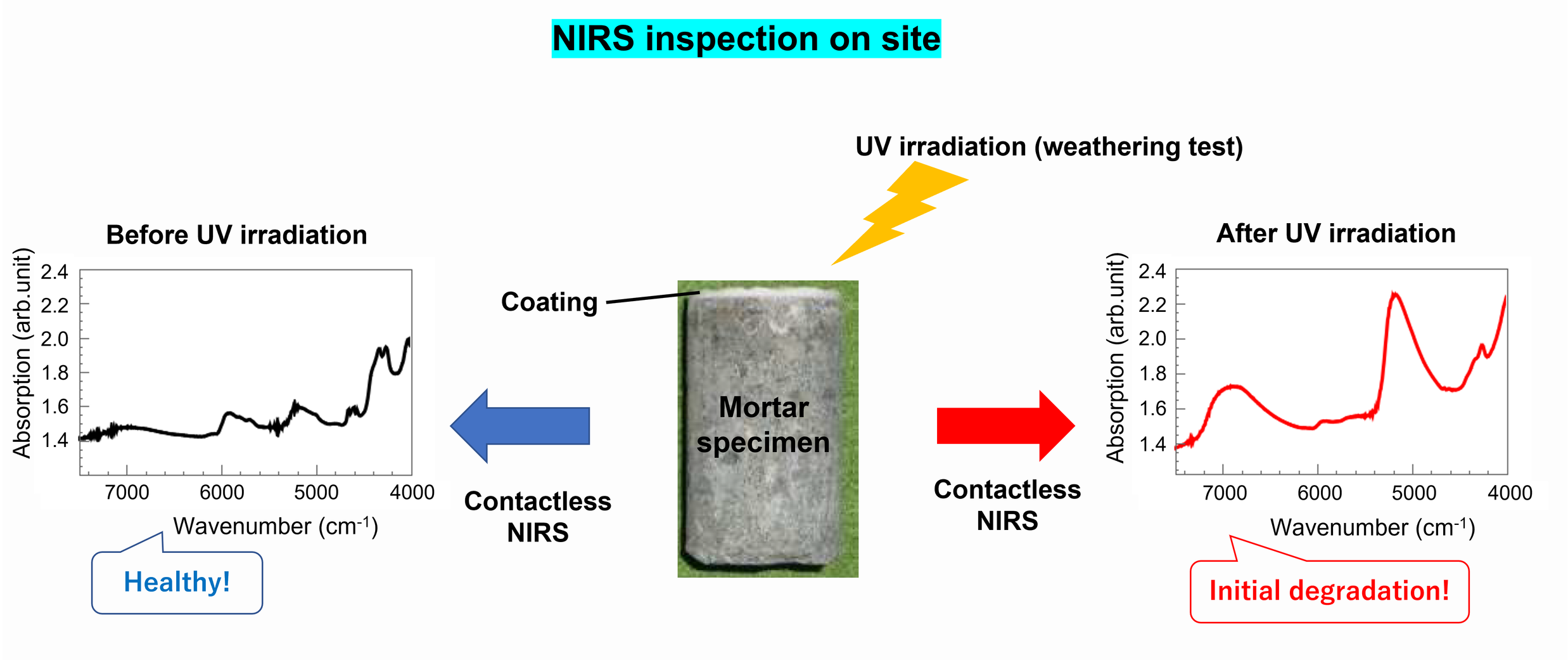

Supplement: S1 Graphical abstract — (TIF) [file pone.0287918.s001.tif]
