# Supplementary material for: Evaluation of near-infrared spectroscopy as a contactless method for health monitoring of resin-based coating materials applied to concrete surfaces
Source: PLoS One. 2023 Jun 28;18(6):e0287918. doi: 10.1371/journal.pone.0287918 (PMC10306184; doi:10.1371/journal.pone.0287918)
Supplement: Attachment — Submitted filename: ReplyToReviewers20230501.docx [file pone.0287918.s002.docx]

Reply to Reviewer’s comments

Reviewer #1: This article presents a good nondestructive method to track the deterioration of coatings applied to concrete surfaces. it would be of interest to many readers and i recommend accepting it after considering the following revisions:
- Lines 197-199: The authors mentioned “Given the annual amount of outdoor UV in the wavelength range of 300–400 nm, based on JIS 9110, these conditions produce deterioration equivalent to that produced by seven years of outdoor weathering”, please mention the JIS or the ASTM standards that the mentioned conditions are equivalent to “seven years of outdoor weathering”

Thank you for your kind comment, we revised the manuscript referring JIS standard. We believe the manuscript became more understandable to readers.

Original: Line 196 “…with the irradiation intensity set at 1500 W/m2 (± 10%). Given the annual amount of outdoor UV in the wavelength range of 300–400 nm, based on JIS 9110, these conditions produce deterioration equivalent to that produced by seven years of outdoor weathering”

Revised: Line 196 ” …with the irradiation intensity set at 1500 W/m^2^ (± 10%) at 300-400 nm in wavelength. According to JIS D0205, average annual UV radiant exposure in Japan is 4500MJ/m^2^ [27], and its composition is 300-400 nm (6.8%) [28], 400-700 nm (44.6%), 700-3000 nm (48.6%). Therefore, the annual radiant exposure in the wavelength range of 300-400 nm is 4500 x 6.8% =306MJ/m^2^. The irradiation of 1500W/m^2^ for 392 h = 392 × 60 × 60 s = 1 411 200 s in this experiment corresponds to 1500 × 1 411 200/ (306 × 10^6^) = 6.9 years outdoor UV exposure.”,

where refs [27] and [28] are “JIS D 205-1987, Test Method of Weatherability for Automotive Parts” and ”CIE publication No.85:1989, Solar Spectral irradiance”, respectively.

- Line 238: please mention the JIS or the ASTM standards used with the “salt-water immersion test”.

As Reviewer 1 indicates, we should have added the information of the standard we used. Since we adopted JSCE (incorporated association, called Japan Society of Civil Engineers) standards, we inserted information as follows,

Original:” 2.6. Salt-water immersion test

Before salt-water immersion,…”

Revised:” **2.6. Salt-water immersion test**

We decided to do salt-water immersion test in order to confirm the protective function of the coating was sustained even after UV irradiation. The procedures of this test including pre-treatment were according to JSCE (Japan Society of Civil Engineers) standards [29]. The analysis of the spatial distribution of chloride ion was then detected by electron probe microanalyzer (EPMA)[30].

Before salt-water immersion,…”

- Lines 296-298: the authors mentioned “The reduction in the absorption intensity of the peak at 2345 nm is particularly stark compared to the peak at 2350 nm, indicating that the C-H bond is more easily broken by UV irradiation”. While in the FTIR section, lines 326-327, it is mentioned “Based on the location of these peaks, this decrease appears to indicate alkyl group cleavage”. I wonder if the results from NIR and FTIR support each others as the alkyl groups cleavage conclusion would reflect more of C-C bonds breakage rather than the C-H bonds cleavage reveled by the NIR. Please elaborate more on the results and explain more if the results from both techniques are supporting each others.

We are sorry for our explanation lead misunderstanding, both NIR and IR results show the cleavage of C-H bond of alkyl group. We revised the FTIR part from

Original Line 326: “Based on the location of these peaks, this decrease appears to indicate alkyl group cleavage”

to

Revised Line370: “Based on the location of these peaks, this decrease appears to indicate a C-H bond cleavage of CH_2_ in the linear alkyl group.”

- Lines 426-427: The authors mentioned “These properties highlight the potential of NIRS as a simple, inexpensive method for inspection of surface coating materials.”. I wonder if the technique can be simply used by engineers in the filed without the need of sending some samples to labs for the SEM-EDX pictures. As it seems to me that the SEM-EDX results were also crucial to confirm the results from both the NIR and FTIR techniques, please comment on this part.

The usage of NIR which Reviewer 1 mentioned is correct. The validity of NIR measurement was confirmed by SEM in this study, therefore, this NIR technique can be used in the field practically without sending the samples to the lab for SEM and other destructive measurements. We added the following sentences.

We revised as follows;

Original:Line388 “…coating film. Near-infrared spectroscopy can be said to…”

Revised: Line452”…coating film. These SEM-EDX images critically showed the validity of the optical non-destructive measurement, i.e. near-infrared spectroscopy can be said to…”

Also we inserted the sentence,

Original: Line 395 “… serious.”

Revised: Line 470“… serious. In the future, it is expected that the NIRS technique can be simply used by engineers in the field without sending samples to labs for SEM-EDX and other destructive measurements.”

- I recommend the authors to conduct extended UV exposure beyond the 396 hours used herein as great extent of degradation might happen after 500 hours for example. Also, it would be better if the author can design a set up in which the samples get exposed to both salty water and UV simultaneously, as this combined effect might alter the coating greatly and it’s more meaningful and mimics exactly what would happen in real life, rather than the effect of UV or salt water alone.

It is assumed that the chemical deterioration level has correlation to the UV exposure time, however, visually radical distraction may occur. Therefore, this comment is reasonable suggestion, and we’d like to think the longer irradiation more than 500 hours. This paper focuses on the simple UV deterioration, and it is the initial study for the inspection of early stage deterioration. For the better understanding for reader, we will try combined effect of UV and salt water in the next paper, whose deterioration scheme should be more complex than this paper.

Reviewer #2: This manuscript reports the results of an interesting research. However, I do not recommend accepting it in the present form due to various problems as described below.

The title and the short title indicate quite different contents. Judging from the title and abstract, the goal of this study is to prove that near-infrared spectroscopy can be used to evaluate the state of deterioration of coatings on concrete surfaces. However, no clear conclusion based on convincing evidence is presented. This is the main flaw of this manuscript.

We revised the short title as follows

“UV does not degrade the mass transfer resistance of concrete coatings.”

Consideration on the following points is also needed.

(1) LL. 206-208: Please specify how the infrared spectrum was used to interpret the near-infrared spectrum. In this paper, near-infrared and infrared spectra are shown, respectively, in nm units and in cm-1 units, so their comparison is difficult. Use of the same units (cm-1) is recommended.

　Thanks to the comment of Reviewer 2, we found our explanation of the reason to use FTIR was not enough. Since the peaks in NIR region tend to overlap each other, which generally makes the description of NIR peak difficult, conventional FTIR of functional group region was used in order to help identifying the specific peaks of NIR. The comparison of NIR and IR spectra was based on the assumption that the major difference between before and after deteriorating reaction in NIR was corresponding to that in IR.

We added the explanation in the beginning part of Section 2.4.

Original: Line 206 “Because the specific absorptions of multiple functional groups may overlap in the near-infrared absorption spectrum, we performed FTIR spectroscopy in the functional group region to enable their identification. Here,…”

Revised: Line 209 “Because the specific absorptions of multiple functional groups may overlap in the near-infrared absorption spectrum, the spectral assignment is difficult without confirmation by conventional methods. Thus we performed FTIR spectroscopy in the functional group region to enable their identification. The spectra of NIR and FTIR were compared, under the assumption that the major difference of spectra in the NIR before and after deteriorating reaction would correspond to that in the IR region. Here,,…”

Also, we revised the horizontal axis of NIR spectra to cm^-1^ unit, as follows,

<Graphical Abstract>

<Fig.1>

<Fig.3>

<Fig. 4>

<Fig. 9>

<Fig. 10>

**Revised: Figure Captions**

Fig. 1. Absorption spectra of the resin-coated mortar specimens in the near-infrared region before UV irradiation. Blue line (a): non-coating; yellow line (b): thin coating; green line (c): normal coating; red line (d): thick coating. Grid lines indicate 4338 cm^-1^ and 4264 cm^-1^.

Fig. 3. Absorption spectra of the resin-coated mortar specimens in the near-infrared region after UV irradiation. Blue line (a): non-coating; yellow line (b): thin coating; green line (c): normal coating; and red line (d): thick coating. Grid lines indicate 4338 cm^-1^ and 4264 cm^-1^.

Fig. 4. FTIR spectrum of the surface of the specimen with normal coating before UV irradiation ((a)blue line) and after UV irradiation ((b)yellow line).

Fig. 9. Absorption spectrum of the base material (blue) (a), main agent (yellow) (b), and topcoat (green) (c), of the acrylic coating in the near-infrared region.

Fig. 10. Schematic diagram of paint layers on a concrete plate modeling: (a-1) severe peeling damage (only the base material remains on the structure), (a-2) moderate peeling damage (the base material and the main agent remain), (a-3) erroneous application (no main agent is included in the resin), and (a-4) an undamaged coating (all layers applied correctly). (b) Absorption spectra of (a-1), (a-2), (a-3), and (a-4) in the near-infrared region (green (a), yellow (b), red (c), and blue (d), respectively).

(2) LL. 281-283: Isn’t the spectrum of the underlying mortar observed not because the resin on the mortar is not even, but because the resin is so thin that near-infrared light is transmitted through it? Or was the uneven resin on the mortar concluded by the photos shown in Fig. 2?

Thank you for your kind comment. There should be a possibility that near-infrared light is transmitted through quite thin coating. Though Fig 2 seems to be uneven coating, we cannot conclude the absence of coating merely by visible appearance. Therefore, we added the sentence,

Line299: “The spectral mixing may also be due to the fact that light has passed through the coating layer of the main agent.”

(3) LL. 292-298: For all the spectra in Fig. 3, strong absorption of the substrate is observed suggesting that most of the coating has been lost for all samples. This is not consistent with the description in the previous paragraph.
The bands at 2345 and 2350 nm cannot be distinguished.

As you mentioned, it is true that the absorption of coating became quite small in Fig. 3. However, Fig. 2 (g), (h) clearly show that the white pigment obviously remains in normal and thick coated samples. This means that the visible inspector on site would judge the coating is healthy, especially in thick coating which has already started to degrade. This is why we propose NIR inspection in this paper.

We really grateful to the indication of our mistyped wavelength value. The wavelength band should be 2345 nm and 2305 nm, not 2350 nm. We are sorry for this mistake. Since the unit in horizontal axis changed from nm to cm^-1^, we deleted the number of nm unit and modified by cm^-1^ unit, in the whole manuscript. We also checked through other mistyped wavenumbers and modified. In addition, we inserted grid lines for corresponding wavenumber in both Fig. 1 and Fig. 3.

(4) LL. 298-301: Stating this leads that there is no basis for discussion.

This part is our assumption of what the reader may simply think just by comparing Fig. 1 and Fig.3, and we tried to explain this assumption is not correct. As you mentioned, the sentences are not clear here, we revised as follows.

Original Line 298: Although it can be assumed that the differences between Fig. 1 and Fig. 3 are because the UV irradiation peeled off part of the coating material, the visual inspection summarized in Fig. 2 indicates that this simple explanation is not valid for all specimens.

Revised Line 328: “At first glance, a reasonable assumption would be that the differences between Fig. 1 and Fig. 3 are because the UV irradiation peeled off part of the coating material. However, the visual inspection summarized in Fig. 2 indicates that this simple explanation seems to be valid only for the thin coating specimen.”

(5) LL.319-320: What is the absorption at 2512 cm-1 assigned to? No significant absorption should be observed in this region usually. Is the absorption due to a component that does not change in the deterioration process and can be used for the standard?

The indication of Reviewer 2 is correct, we should have mention it. We added the information.

Original Line 319: “As measurement was conducted using diffuse light, the intensity of each peak was standardized to that of the peak at 2512 cm^-1^.”

Revised Line 354: “As measurement was conducted using diffuse light, the intensity of each peak was standardized to that of the peak at 2512 cm^-1^, where no significant absorption should be usually observed.”

(6) LL. 320-323: Please assign the bands clearly.

We assigned the C-O bond of ester, C=O bond, and C-H bond of alkyl from the peak positions, however, we don’t have the precise name of molecule used in acrylic resin emulsion, since this is the industrial secrets. We used the daily used agent in the actual construction site in Japan because we wanted to apply the NIR inspection in the status close to real site. We cannot conclude the bands for which part of the molecule, however, we can mention the name of product instead. We added the SDS product number in Section 2.1. (We also added the Acknowledgement for the company to tell us the main component of the product.)

(7) LL.324-326: Please clarify which bands show similar changes by quantitative evidence.

Since the sensitivity of each wavenumber is different between NIR and IR spectrometers, the change of peak intensity cannot be compared directly. Here, we added the comparison of the ratio of two peaks, i.e., 4338 cm^-1^ peak intensity with respect to that of 4264 cm^-1^ in NIR, and 2950 cm^-1^ peak intensity with respect to that of 2870 cm^-1^ in IR.

We inserted the sentences as follows,

Line 362: “The peak intensity ratios for 2950 cm^-1^ to 2870 cm^-1^ in FTIR (normal coating) were 1.15 (before UV irradiation) and 0.638 (after UV irradiation), where the peak intensity was measured from the intensity at 3130 cm^-1^, the skirt of the peak. While, the peak intensity ratio for 4338 cm^-1^ to 4264cm^-1^ in NIR (normal coating) were 0.966 (before UV irradiation) and 0.608 (after UV irradiation), where the peak intensity is measured from the intensity at 4480 cm^-1^, the skirt of the peak. Although the changes of peak intensity cannot be compared directly since the sensitivity of each wavenumber is different between NIR and IR spectrometers, the peak intensity ratios for 4338 cm^-1^ to 4264cm^-1^ in NIR and that for 2950 cm^-1^ to 2870 cm^-1^ in IR were reduced to about 63% and 55% respectively, due to UV irradiation.”

(8) LL.327-329: Please indicate what kinds of reaction you expect.

The reaction we expect is the C-H bond cleavage of CH_2_ in linear alkyl group, therefore, we added some words as follows.

Original Line 326: “Based on the location of these peaks, this decrease appears to indicate alkyl group cleavage.”

Revised Line 370: “Based on the location of these peaks, this decrease appears to indicate a C-H bond cleavage of CH_2_ in the linear alkyl group.”

(9) The descriptions in Sections 3.3 to 3.5 have little relation to the results of the near-infrared spectroscopy. Their relation to the main target of this manuscript cannot be understood.

As we mentioned in Section 1 (Line 107-112 in previous version), Sections 3.3 to 3.5 are reference tests with conventional methods for backing NIR data. The results in Sections 3.3 to 3.5 explain that the extent of deterioration by UV irradiation is slight, and the protection function is maintained.

We added explanation,

Line 402: “Similar to Section 3.3, the conventional inspection of the salt-water immersion test is performed as a reference test of NIR spectroscopy, which proves the protection from the external substance both before and after the UV light irradiation.”,

and

Line 423 “The micro structural information was obtained as supplemental data to explain the NIR measurement.”,

in the beginning part of Section 3.4 and Section 3.5, respectively.

(10) Only the obvious conclusion that needs no discussion is stated in section 3.6.

The aim of Section 3.6 is to show one of the possible applications of NIR technique in the inspection site, not merely showing the obvious results.

(11) Followings should also be revised.
･ There are many spelling mistakes, grammatical mistakes, and unnatural English expressions.

We are sorry for this, and we have already gotten English proofreading in the revised manuscript.

･ It is necessary to indicate what JIS is.

JIS stands for “Japanese industrial standards”. We added the formal name of JIS in the first appearance of JIS.

Revised Line 159: “The series of coatings, base material, main agent, and topcoat used in this study meet the requirement of JIS (Japanese Industrial Standards) A6909 [25].”

･ L.250: Since the SI system of units does not allow the use of M for molarity, it is recommended to use mol L-1.

Thank you for the suggestion, we revised M to mol/L.

Line 262: “… 0.5 mol/L …”

･ Figs. 1, 3, 4, 9, and 10: Label for horizontal axis should be below the spectrum. It is easier to distinguish the spectra by not only distinguishing them by color, but also by adding symbols such as a, b, c, ･･･.

Thank you for your comment. We changed the axis label positions and added indicating symbols to these Figures.

Reviewer #3: This paper reports the results of a study to demonstrate that NIR spectrometry can be used to inspect defects in protective coatings of concrete, including those due to ageing. The experimental design was properly set up and executed; the results are convincing and well discussed. I therefore recommend publication of this paper with some minor modifications:

- line 74: the authors mention pharmacy as a field of use of NIRS, whereas there are many others, such as synthetic chemistry (e.g. polymers), petrochemistry, food processing, etc. This passage should be amended accordingly.

Thank you very much for your kind advice, we added the reference of synthetic chemistry, petrochemistry, and food processing, in which we were very interested.

Original Line 72: At present, simple and immediate measurement methods are required in the construction field. Near-infrared spectroscopy (NIRS), a technique employed extensively in the field of pharmacy; in addition, it is used for real-time monitoring of the uniformity of the concentration of active ingredients in tablets as well as coating thickness of tablets [16–18].

Revised Line72: “At present, simple and immediate measurement methods are required in the construction field. Near-infrared spectroscopy (NIRS), a technique employed extensively in the fields of pharmacy [16-18], synthetic chemistry [19], petrochemistry [20], and food processing [21][22] is a powerful tool for chemical analysis during and after production, and even useful for real-time monitoring.”

The added references [19]-[22] are followings.

19. Hammond J., Kellam B., Moffat A. C., Jee. R. D., Anal. Commun., Non-destructive real-time reaction monitoring in solid phase synthesis by near-infrared reflectance spectroscopy –esterification of a resin-bound alcohol, 1999, **36**, 127-129.

20. Macho. S., Larrechi M. S., Near-infrared spectroscopy and multivariate calibration for the quantitative determination of certain properties in the petrochemical industry, TrAC., vol 21, no. 12, 2002.

21. Grassi S., Alamprese C., Advances in NIR spectroscopy applied to process analytical technology in food industries, Curr. Opin. Food. Sci., 2018, **22**, 17-21.

22. Nieto-Ortega S., García S.M., Melado-Herreros Á, Foti G., Olabarrieta I., Roger J.M., Food. Bioproc. Tech., (2023) 16:881-896.

- line 78: the authors write that each functional group absorbs at a unique wavelength, but this is not entirely true. True, the absorption peaks are quite sharp in the MIR, but they span several wavelengths.

Thank you very much for your kind suggestion. We revised manuscript, following to the useful advice.

Original Line77: “In the mid-infrared region used in FTIR analysis, each functional group absorbs infrared light at a unique wavelength (functional group region) such that it can be identified based on an absorption wavelength peak.”

Revised Line 76: “In the mid-infrared region used in FTIR analysis, each functional group absorbs infrared light at several characteristic wavelengths (functional group region), appearing as sharp peaks, therefore it can be identified based on absorption wavelengths.”

- line 93: change stategic by strategic

Thank you for your comment, we revised as follows,

Original Line 93:” surface of concrete. Early detection of the deterioration makes the stategic maintenance

Revised Line 93:” surface of concrete. Early detection of the deterioration makes the strategic maintenance”

- lines 281 - 285: the discussion suggests that the observed spectral mixing comes from lack of coverage of the protective agent. But the mixing may also be due to the fact that the light has passed through several layers of product. The few sentences in this passage could be changed in this sense, without changing the conclusion.

This comment is very important, we grateful to it. We revised the manuscript as follows,
Original Line 283: “…peaks for both the mortar and the resin could be observed. Thus, we suggest that this behavior…”

Revised Line 299: “…peaks for both the mortar and the resin could be observed. The spectral mixing may also be due to the fact that light has passed through the coating layer of the main agent. Thus, we suggest that this behavior…”
