# Supplementary material for: Evaluation of near-infrared spectroscopy as a contactless method for health monitoring of resin-based coating materials applied to concrete surfaces
Source: PLoS One. 2023 Jun 28;18(6):e0287918. doi: 10.1371/journal.pone.0287918 (PMC10306184; doi:10.1371/journal.pone.0287918)
Supplement: Attachment — Submitted filename: Reply to Reviewers 20230607.docx [file pone.0287918.s003.docx]

**Reply to Reviewers**

Reviewer #4: Manuscript Number: PONE-D-22-27578R1

Evaluation of near-infrared spectroscopy as a contactless method for health monitoring of coating materials applied to concrete surfaces

The work proposes using NIR spectroscopy for characterization of coating materials applied to concrete. I suggest the authors to present what are these materials (resin-based materials) in the abstract. Also, perhaps this can be added to the title, to clarify to the audience the materials investigated. This would be important, considering the general knowledge (and also what the authors mention in the introduction) about the signals in the NIR spectra that are related to organic material.

-Thank you for your kind suggestions. We inserted the words “resin-based” and “organic resin-based” for title and abstract.

Previous Title: **Evaluation of near-infrared spectroscopy as a contactless method for health monitoring of coating materials applied to concrete surfaces**

Revised Title: **Evaluation of near-infrared spectroscopy as a contactless method for health monitoring of resin-based coating materials applied to concrete surfaces**

Previous Line 27: simple inspection for health monitoring of coating materials.

Revised Line 27: simple inspection for health monitoring of organic resin-based coating materials.

Material and methods

Section 2.1. Please clarify the total number of samples investigated. Also, please clarify the number of samples used for calibration and validation, as it is mandatory to have an external validation dataset.

-We prepared a single sample for each measurement, since the space of the sample holder of the UV irradiation equipment is limited. We revised the manuscript as follows. We also added the information for Section 3.6, without UV irradiation.

Added line 156: Totally, 2 (before/after UV irradiation) × 4 (coating thickness) × 5 (kinds of measurements, in case of NIRS, FTIR, Permeability test, Salt-water immersion test, SEM) = 40 samples were prepared.

Added line 180: The total number of the samples for the peeling characterization was four.

In this paper, we did not use statistical method such as chemometrics yet. Since this study is the fundamental investigation to clarify the ability of deterioration detection by NIRS, when the degree of the deterioration is slight.

Results and discussion

As the authors present, the main test for the degradation was the water-permeability, and water has a signal on NIR spectra. Hence, it seems that the main determination is actually the presence of water in the coatings. I suggest the authors to clarify this information, and enhance the discussion, considering this aspect.

-It is well known that the protective organic paintings are deteriorated by mainly UV irradiation during weathering test. This photo-oxidative deterioration decreases water protection ability; therefore the permeability of organic coatings generally increases with longer period of exposure to UV irradiation [“Influence of the photo-oxidative degradation on the water barrier and corrosion protection properties of polyester paints”, F. Deflorian, L. Fedrizzi, P.L. Bonora, Corrosion Science, vol. 38 (1996) 1697-1708]. [“Influence of coating system composition on moisture dynamic performance of coated wood,” Jan Ekstedt, Journal of Coatings Technology vol. 75 (2003) 27–37].

In this study, it was found that the chemical deterioration due to moderate UV irradiation did not affect permeability yet. Since we haven’t investigated the physical degradation such as cracks and peelings caused by this chemical deterioration with more harsh UV irradiation condition, it will be confirmed in the next study.

As the Reviewer suggests, the absorption peak originated from “-OH of water” clearly can be seen in Fig 1 and Fig 3. The reason why these the peak intensity different between before and after UV irradiation, may be because large amount of water was applied to the samples by the shower during the weathering test or because some chemical reaction changed the environment around -OH bond. We think that the former possibility is reasonable, since all of the peaks at around 7000 cm^-1^ and 5200 cm^-1^ radically and similarly enhanced in thin, normal, and thick coating samples in Fig. 3.

As our future task, we would like to investigate the easier method to evaluate the deterioration level, which is directly related to protection ability of the coating materials, by carefully referring results of more through comparison experiments.

We added the following sentences to the manuscript,

Line 234: It is well known that the protective organic paintings are deteriorated by mainly UV irradiation during weathering test. This photo-oxidative deterioration decreases water protection ability; therefore, the permeability of organic coatings generally increases with longer period of exposure to UV irradiation [32][33].

Line 355: It is noteworthy that the prominent braod peaks of water appeared in Fig. 3 at around 5200 cm^-1^ and 7000 cm^-1^, which may be because the large amount of water was applied to the samples by the shower during the weathering test.

Previous works have used NIRS to determine the mechanical properties of different materials. However, the mechanical properties are correlated to the composition of organic materials. Hence, I hereby list a few works on the subject (as a suggestion, the authors may find others) that may provide support for the discussion, stating that the main determination is actually the composition or presence of water, and it is correlated to the coating materials deterioration.

VIS–NIR spectroscopy as a process analytical technology for compositional characterization of film biopolymers and correlation with their mechanical properties - https://doi.org/10.1016/j.msec.2015.06.029

Application of VIS/NIR spectroscopy for estimating chemical, physical and mechanical properties of cork stoppers. Wood Sci Technol 48, 811–830 (2014). https://doi.org/10.1007/s00226-014-0642-3

Comparison of Methods for Estimating Mechanical Properties of Wood by NIR Spectroscopy - https://doi.org/10.1155/2018/4823285

-This study is the previous stage of the estimating physical/chemical properties quantitatively. Therefore, only qualitative discussions are written. However, thank you very much for suggesting us to try the quantitative prediction of the deterioration degree. We will prepare many samples enough for the statistical calibration such as PLS in the next study. At the first onset, we have just prepared another new UV irradiation equipment and made new sample holders which we can put more samples at once. We added the sentence as follows:

Line 78: Also, visible and NIR spectroscopy is often used for prediction of physical and mechanical properties, supported by chemometrics procedures [23][24][25].

I suggest to change the short title presented in line 4, page 1/58

-We revised the short title, following to the advice of the Reviewer.

Previous short title: UV does not degrade the mass transfer resistance of concrete coatings.

Revised short title: Moderate UV irradiation does not degrade the mass transfer resistance of concrete covered with organic resin coatings.
